# Selenium, Zinc, and Copper Status of Vegetarians and Vegans in Comparison to Omnivores in the Nutritional Evaluation (NuEva) Study

**DOI:** 10.3390/nu15163538

**Published:** 2023-08-11

**Authors:** Lea Klein, Christine Dawczynski, Maria Schwarz, Maria Maares, Kristin Kipp, Hajo Haase, Anna P. Kipp

**Affiliations:** 1 Institute of Nutritional Sciences, Friedrich Schiller University, 07743 Jena, Germany; lea.klein@uni-jena.de (L.K.); schwarz.maria@uni-jena.de (M.S.); 2 Junior Research Group Nutritional Concepts, 07743 Jena, Germany; 3 TraceAge—DFG Research Unit 2558, 07743 Potsdam-Berlin-Jena-Wuppertal, Germany; maares@tu-berlin.de (M.M.); haase@tu-berlin.de (H.H.); 4 Department of Food Chemistry and Toxicology, Technische Universität Berlin, Straße des 17. Juni 135, 10623 Berlin, Germany; 5 Department for Pediatrics, Sophien- and Hufeland Klinikum, Hospital Weimar, 99425 Weimar, Germany; k.kipp@klinikum-weimar.de

**Keywords:** selenium, zinc, copper, vegans, plant-based diets

## Abstract

Plant-based diets usually contain more nutrient-dense foods such as vegetables, legumes, whole grains, and fruits than a standard Western diet. Yet, the amount and especially the bioavailability of several nutrients, such as trace elements, is supposed to be lower in comparison to diets with consumption of animal-derived foods. Based on this, the Nutritional Evaluation (NuEva) study (172 participants) was initiated to compare the trace element status of omnivores, flexitarians, vegetarians, and vegans. Serum selenium, zinc, and copper concentrations and biomarkers were evaluated at baseline and during a 12-month intervention with energy- and nutrient-optimized menu plans. The implementation of optimized menu plans did not substantially influence the status of trace elements. At baseline, serum selenium biomarkers were lower in vegetarians and vegans compared to omnivores and flexitarians. The zinc intake of vegetarians and vegans was significantly lower compared to omnivores, whereas the Phytate Diet Score was increased. Accordingly, total serum zinc concentrations were reduced in vegans which was, however, only significant in women and was further supported by the analysis of free zinc. Regarding copper status, no differences were observed for total serum copper. Overall, we identified selenium and zinc as critical nutrients especially when maintaining a vegan diet.

## 1. Introduction

In recent years, plant-based diets are becoming increasingly popular in Western countries. Over the last decade, the number of vegetarians and vegans has increased from 1.6% and <0.5%, respectively, to 5.4–10% and 1–2% of the German population [1,2,3,4]. This prevalence is comparable to other European countries [5,6] and the US [7]. Those dietary patterns are characterized by omitting selected animal foods such as fish and meat (vegetarian) or total avoidance of animal foods and additives (vegan). Besides ethical and environmental motivations, potential health benefits of a plant-based diet contribute to the increased popularity [8]. To better characterize the benefits but also risks associated with a plant-based diet the Nutritional Evaluation (NuEva) study has been conducted. This study was designed to investigate the influence of dietary patterns on nutrient supply and cardiovascular risk factors. The NuEva study included four different dietary patterns, namely a traditional Western diet with daily meat or sausage intake (omnivores), flexitarians (meat or sausage intake once or twice per week), as well as vegetarians and vegans. Based on the baseline data of the subjects, practical nutritional concepts were developed provided as energy- and nutrient-optimized menu plans to cover potential nutrient deficits that might result from the different dietary patterns. The study participants followed the menu plans for 12 months [9].

In particular, avoidance of meat and high consumption of whole grains, vegetables, and fruits have been associated with a lower risk of developing cancer, cardiovascular disease, and type 2 diabetes [10,11,12,13]. One major characteristic of a plant-based diet is the high intake of fiber, which is most often below the recommendation of 30 g per day in omnivores (median: 24.4 g/d ranging from 9.5 to 54.6 g/d), but clearly above this value in vegetarians (median: 30.3 g/d ranging from 6.4 to 85 g/d) and vegans (median: 36.8 g/d, ranging from 14.8 to 115 g/d) [14]. However, avoidance of certain food groups does not only increase the intake of food components such as fiber but also decreases the intake of others. In addition to meat and fish, milk is also an important food source of trace elements, such as iron, iodine, zinc, and selenium. In addition to lower concentrations, bioavailability is also most often reduced from plant-derived foods [15]. This is well-established for iron as the heme iron found in animal-derived foods has a higher bioavailability than non-heme iron from plant-based foods [16], but also for zinc which is tightly bound by phytate, substantially reducing its bioavailability [17]. Based on the described changes in micronutrient intake, we focused on three trace elements, namely selenium, zinc, and copper. (i) Selenium was chosen because it is mainly supplied by animal-derived foods in Europe where its intake is most often below adequate values in the general population [18]. (ii) Zinc is one of the major examples of nutrients with reduced bioavailability induced by high amounts of fiber and phytate. Furthermore, for zinc, values of recommended intake are often not met, especially in young women at reproductive age [19,20]. (iii) The intake of copper might be higher in vegetarians/vegans in comparison to omnivores due to the consumption of high amounts of legumes, nuts, and seeds [21]. However, copper bioavailability might also be reduced by fiber. Besides this, it is well established that copper and zinc are tightly connected in terms of absorption. Changes in copper/zinc ratios are often more reliable risk predictors for diseases than single trace element concentrations [22]. To characterize the status of these three trace elements not only circulating serum concentrations are analyzed, but also functional biomarkers are used. In the case of selenium, this includes activity and/or concentration of the circulating selenoproteins glutathione peroxidase (GPX) 3 and selenoprotein P (SELENOP), respectively. Ceruloplasmin (CP) is a copper-dependent ferroxidase that mainly contributes to the amount of copper in serum [22]. In addition, free copper, which potentially represents the more toxic copper, was determined. For zinc, protein-based biomarkers are scarce, but recently the amount of circulating free zinc has been discussed to function as more reliable biomarker than total zinc concentrations [23]. In addition, Zinc and Phytate Diet Scores have been developed and validated to predict serum zinc concentrations [24].

Accordingly, we aimed to characterize the status of selenium, zinc, and copper of NuEva study participants with a high intake of animal-based foods (Western diet), flexitarians, vegetarians, and vegans in Germany. The effect of optimized daily menu plans for the different dietary patterns on the trace element supply was analyzed. The menu plans provided zinc and copper in accordance with the reference values of the German Nutrition Society (D-A-CH reference values). Due to a lack of data on the selenium content in foods in the used database (Bundeslebensmittelschlüssel^®^), it was not possible to adjust the menu plans for selenium intake.

## 2. Materials and Methods

### 2.1. Participants

In summer/autumn 2018, healthy women and men between 18 and <70 years following an omnivore, flexitarian, vegetarian, or vegan diet for at least one year before enrollment were recruited. The adherence to one of the four dietary patterns was assessed by an interview before enrollment and proofed by the dietary protocol before the first study visit. The omnivorous diet was comparable to a Western diet, which is defined by a regular (daily) consumption of meat and sausages, while the flexitarian diet only included occasional consumption (1–2 × per week) of meat and sausages. In both groups, consumption of fish was not limited, but also not a prerequisite. Vegetarians were defined as people consuming dairy products and eggs in addition to plant products, whereas vegans were defined as people living solely on food of plant origin. Further exclusion criteria from the study have been published elsewhere [9]. In the present investigation, only the subjects who completed all blood samplings (baseline and after 3, 6, 9 and 12 months) of the NuEva intervention period were examined. Three vegans had to be excluded from the statistical analysis because they had exceedingly high selenium concentrations and were considered as outliers after performing Grubbs’ test. In addition, one subject diagnosed with Wilson’s disease was excluded, as this hereditary disease significantly affects copper metabolism. In total, 40 omnivores, 47 flexitarians, 45 vegetarians and 40 vegans were examined. 

### 2.2. Study Design and Diets

The NuEva study aimed to reveal the impact of common nutritional habits on health status and disease risk. The NuEva concept was designed to improve nutritional behavior based on three columns: (1) personalized menu plans that are adapted to individual needs and requirements, (2) regular nutritional counseling units including feedback, and tracking, and (3) various incentive strategies. 

The NuEva study was designed as a prospective, non-randomized, monocentric intervention study in parallel design with four arms (omnivore diet, flexitarian diet, vegetarian diet, and vegan diet). The menu plans are in accordance with the hallmarks of the studied dietary patterns and are further characterized by:

1. Adequate amounts of energy derived from carbohydrates, protein, and fat in accordance with the guidelines of the German Society of Nutrition [25];
2. Defined intake of saturated fatty acids (SFA, <10% of daily energy), monounsaturated fatty acids (MUFA, >10% of daily energy), PUFA (~10% of daily energy), and at least two grams of alpha-linolenic acid per day, e.g., through linseed oil (5–10 mL per day between study month 3 and 12);
3. Encouraged consumption of vegetables, fruits, and cereals;
4. Intake of >40 g dietary fiber per day based on the MoKaRi concept [26];
5. Salt (maximum of 6 g per day) and sugar reduction (maximum of 50 g per day);
6. Reduced intake of highly processed, calorie-rich, low-nutrient foods (fast foods, convenience products);
7. Optimized intake of vitamins, minerals, and trace elements by commercially available foods, considering the seasonal availability of vegetables and fruits.

The menu plans were adapted to participant’s individual energy requirements considering their age, gender, and level of physical activity. Selenium intake of the menu plans is unknown and cannot be estimated because selenium concentrations in foods are only poorly recorded in databases.

The NuEva study started with a run-in period to assess and document the dietary habits of the study participants using a dietary record over five days. To investigate the nutritional and health status of the subjects, a single blood draw and collections of fecal and 24 h urine samples were performed before the start of the study in the screening phase. During the intervention phase (12 months) a checkup with anthropometric, pulse, and blood pressure measurement as well as a blood sampling was performed every three months. In addition, the subjects received nutrient-optimized daily menu plans. Further information on the study design is accessible in the published study protocol [9]. 

The study protocol was reviewed and approved by the Ethical Committee of the Friedrich Schiller University Jena on the 24 April 2018 (number: 5504-03/18). The NuEva study was registered before launching (Clinical-Trials.gov Identifier: NCT03582020).

### 2.3. Nutrient Intake

The run-in phase of the NuEva study included a full self-report of the individual dietary intake over 5 days. The dietary record was based on the template “Freiburger Ernährungsprotokoll”, which was provided by PRODI^®^ version 6.4 (Nutri-Science, Stuttgart, Germany) and included common foods and usual portion sizes. The template was adapted to the NuEva study by adding foods that are favored in vegetarian and vegan diets, such as tofu, vegan yogurt alternatives and plant drinks, soy products, seitan, tempeh, maple, and agave syrup (further foods could be added individually). Foods that were not contained in PRODI^®^ were created and the nutritional information was taken from the packaging (including fortification with, e.g., vitamin B12, calcium). The daily energy and nutrient intakes were calculated using the software package PRODI^®^. The nutrient intake from supplements was additionally queried. Due to a lack of data on the selenium content in foods in the used database (Bundeslebensmittelschlüssel, Federal Ministry of Food and Agriculture, 53107 Bonn, Germany), only information about the zinc and copper intake was available and is presented here. 

### 2.4. Zinc and Phytate Diet Score

Trame et al. developed a biochemically validated questionnaire to calculate a Zinc and Phytate Diet Score [24]. The questionnaire includes 18 food items, of which the zinc and phytate contents were calculated using the nutrition and calorie table of Elmadfa et al. [27] and the overview of Schlemmer et al. [28], respectively. The questionnaire covers the period of the last 6 months as well as the frequency of consumption, the portion size or amount and the usage of zinc supplements. The frequency of consumption was assigned a factor of 1 to 5 (1 = never, 2 = 2–3 × per month, 3 = 1 × per week, 4 = 2–4 × per week, 5 = daily) and portion size was assigned a factor of 1 to 3 (1 = small, 2 = medium, 3 = large). The calculation of the Zinc or Phytate Diet Score is based on the following formula:

Zinc or Phytate Diet Score = frequency index × portion index × zinc or phytate content [mg].

In this investigation, the Zinc and Phytate Diet Scores were calculated using 164 completed dietary records of the Run-In period. Dietary records were missing from 11 subjects and in addition, one dietary record had to be excluded due to underreporting. Food frequency and portion size were derived from the dietary records and inserted into the 18-item questionnaire of Trame et al. [24]. For frequency assessment, the duration of the protocol (5 days) was considered for each food category. Consequently, no conclusions could be drawn about the monthly consumption (2–3 × or 1 × per month) of the food items. The dietary records included the portion of foods in grams, which allow calculation of the portion size. During the Run-In period, none of the subjects reported using zinc supplements.

### 2.5. Laboratory Measurements

#### 2.5.1. Total Serum Trace Element Analysis

Fasting peripheral venous blood samples were collected, fully coagulated at room temperature, and centrifuged (10 min, 2762 g, 4 °C) for separation of serum. Before analysis, serum samples were stored at −80 °C. Total trace element levels were measured by X-Ray fluorescence, using a bench-top total reflection X-ray fluorescence (TXRF) spectrometer (S4 T-StarTM, Bruker Nano GmbH, Berlin, Germany). As internal standard 1 mg/mL Gallium (Thermo Fisher Scientific, Kandel, Germany) was used. In total, 10 μL of each sample was placed on quartz glass carriers and dried at 40 °C. Samples were measured in duplicates for 750 s. For quality control, reference material RECIPE^®^ ClinChek^®^ serum control lyophilized 1 (Ref. 8880, Lot 544) was measured in duplicate frequently (in every measuring process). For a classification of the serum trace element levels, the reference values according to *Labor und Diagnose* were used. Reference ranges indicating normal serum concentration of selenium, zinc and copper were defined as 50–120 µg/L, 600–1200 µg/L, and 580–1690 µg/L, respectively [29]. The reference range for copper differs between men and women, with the reference range being higher for women. Therefore, the lowest value for men and highest value for women was chosen.

#### 2.5.2. Free Zinc and Free Copper Analysis

Free serum zinc levels were determined using the low-molecular-weight fluorescent sensor Zinpyr-1 (Santa Cruz biotechnology, Dallas, TX, USA) as described [23]. To this end, 20 μL of serum, prediluted in assay buffer (1:10) and stored at −80 °C, was added to 80 μL prewarmed assay buffer containing Zinpyr-1 (final concentration 0.05 μM). Free zinc was determined on the basis of the fractional saturation of Zinpyr-1 using 15 μL EDTA or 800 μM ZnSO_4_ to induce a maximal and minimal fluorescence signal of the sensor, respectively [23]. The dissociation constant (Kd) for the Zinpyr-1–zinc complex of 0.7 nM was then used for the calculation of free serum zinc concentrations.

The concentration of free copper was quantified by a fluorometric method using a copper-binding fluorescent peptide (FP4) based on the dansyl peptide 4 (DP4) by Young et al. [30] but coupled to carboxyfluorescein as fluorophore. Serum samples were pre-diluted (1:20) in assay buffer and stored at −80 °C. An aliquot of 20 µL was added to 80 µL assay buffer containing FP4 (final concentration 10 nM) at room temperature. Free serum copper concentrations were calculated according to Grynkiewicz et al. [31], determining the maximal and minimal fluorescence signal of FP4 after addition of 2 mM EDTA or 2.2 mM CuSO_4,_ respectively, and using a dissociation constant for the FP4-copper-complex of 0.38 pM. 

#### 2.5.3. GPX Activity

As described previously [32], a NADPH-consuming glutathione reductase-coupled assay was applied for assessment of GPX activity (glutathione reductase obtained from Roche Diagnostics, Basel, Switzerland), while hydrogen peroxide (Merck, Darmstadt, Germany) was used as substrate. Here, 14 μL of diluted serum (1:5) was used and absorbance was measured in triplicate. GPX activity is expressed as U/L. Due to a turbidity in the serum of four samples, the enzyme activity could not be reliably determined, and the measured values of these subjects were not included in the evaluation of the results.

#### 2.5.4. Ceruloplasmin Activity

As described previously [33] serum ceruloplasmin (CP) was measured by its copper-dependent oxidase activity using o-dianisidine dichloride (VWR, Radnor, PA, USA) as substrate. O-dianisidine dichloride is oxidized in the presence of oxygen by CP to an undetermined chromophore that can be measured spectrophotometrically. Here, 5 µL of diluted serum (1:2) was used and CP activity was determined as a single measurement after 10 min (sample background) and as a triplicate after 60 min (sample reaction) on separate 96-cavity microtiter plates using a microplate reader (Synergy H1, BioTek, Bad Friedrichshall, Germany). CP activity is expressed as U/L. Due to a turbidity in the serum of four samples, the enzyme activity could not be reliably determined, and the measured values of these subjects were not included in the evaluation of the results.

### 2.6. Statistical Analyses 

The statistical analysis was performed using GraphPad Prism 8. Statistical differences between men and women were tested by a Mann–Whitney U-test. If sex had a significant influence, the statistical analysis was performed and reported for men and women separately. If the data of the four groups followed a normal distribution (tested with Shapiro–Wilk), ordinary one-way ANOVA (using Benjamini–Hochberg correction) was applied for non-paired samples. Otherwise, a Kruskal–Wallis test (using Benjamini–Hochberg correction) was used. Repeated measures ANOVA (using Benjamini–Hochberg correction) was used for matched samples (comparison of time points) when a normal distribution existed. If the groups did not follow a normal distribution, a Friedman test (using Benjamini–Hochberg correction) was applied. Correlation analysis was performed based on Spearman correlation. Statistical significance is demonstrated by *p*-values smaller than 0.05.

## 3. Results

### 3.1. Characteristics of Study Participants

A total of 258 subjects participated in the NuEva Study out of which 70% were women and 30% were men. The subjects’ age ranged between 18 and 69 years. The characteristics and anthropometric data of the entire study collective have been published previously [14]. Here, a separate analysis of subjects who completed all blood samplings of the NuEva intervention period was performed. A total of 176 subjects were examined (72% women, 28% men) and for the final analysis, data from 172 subjects were included, because four subjects had to be excluded (see Section 2.1). Of these, 40 subjects were omnivores, 47 were flexitarians, 45 were vegetarians, and 43 were vegans, with the age of the vegan group being significantly lower compared to the omnivores (*p* = 0.03). In men, the weight did not differ significantly between the four studied diet groups. The flexitarian and vegan women had significantly lower body weight compared to the omnivores (*p* < 0.001). Furthermore, the Body Mass Index (BMI) of flexitarians and vegans was significantly lower compared to omnivores (*p* < 0.05) (Table 1).

### 3.2. Baseline Serum Selenium Status

**Figure 1:**
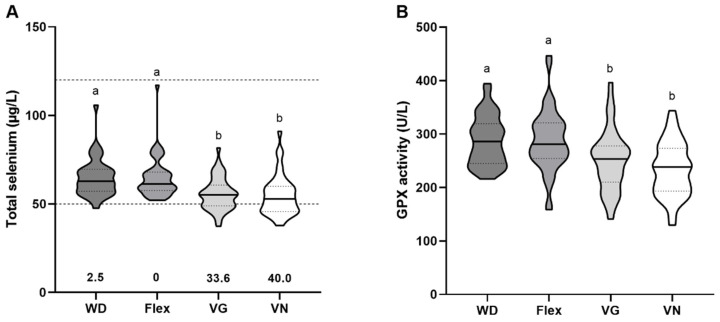
Analysis of selenium biomarkers at baseline. (**A**) Total selenium concentrations in the serum of omnivores (WD, *n* = 40), flexitarians (Flex, *n* = 47), vegetarians (VG, *n* = 45), and vegans (VN, *n* = 40) were determined using total reflection X-ray fluorescence spectroscopy (TXRF). The dashed lines indicate the reference range for serum selenium concentrations, with the numbers below the violin plots representing the percentage of subjects below this threshold. (**B**) Enzymatic activity of glutathione peroxidases (GPX) (WD, *n* = 38; Flex, *n* = 47; VG, *n* = 43; VN, *n* = 40) was measured by colorimetric assay. The results are shown as violin blot with median and 25- and 75-quartile range (indicated as dotted lines). Diet groups that do not share indices (a,b) differ significantly (Kruskal–Wallis test followed by Benjamini–Hochberg correction (*p* < 0.05)).

The serum selenium concentrations varied between the four studied groups with the highest serum selenium concentrations in omnivores with a median of 62.85 μg/L and flexitarians at 61.37 μg/L, whereas the selenium levels of vegetarians and vegans were significantly lower at 55.12 μg/L and 52.84 μg/L (*p* < 0.001), respectively. Overall, the median serum selenium levels of all groups were in the lower third of the reference range. While almost none of the omnivores (2.5%) and flexitarians had selenium values below the limit of 50 µg/L, this occurred relatively frequently in the vegetarian (33%) and vegan groups (40%) (Figure 1A). In accordance with the serum selenium concentration, the serum GPX activities of the vegetarians and vegans were significantly lower with median values of 253.10 U/L and 238.40 U/L in comparison to the omnivores and flexitarians with 285.90 U/L and 281.00 U/L, respectively (Figure 1B) (*p* ≤ 0.006). 

### 3.3. Baseline Zinc Status

**Figure 2:**
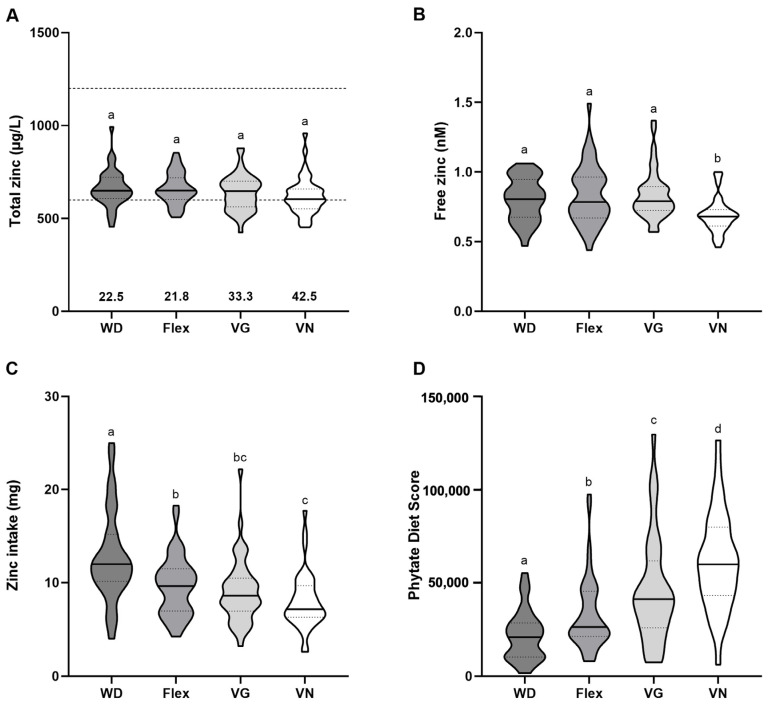
Analysis of zinc biomarkers at baseline. (**A**) Total zinc concentrations in the serum of omnivores (WD, *n* = 40), flexitarians (Flex, *n* = 47), vegetarians (VG, *n* = 45), and vegans (VN, *n* = 40) were determined using total reflection X-ray fluorescence spectroscopy (TXRF). The dashed lines indicate the reference range of zinc in serum, with the numbers below the violin plots representing the percentage of subjects below this threshold. (**B**) Free serum zinc levels were quantified by a fluorometric method using the low-molecular-weight fluorescent sensor Zinpyr-1. (**C**) Daily zinc intakes were calculated from baseline dietary records conducted over 5 days (WD *n* = 37, Flex *n* = 46, VG *n* = 39 and VN *n* = 38). (**D**) Phytate Diet Scores of baseline dietary records were calculated based on the questionnaire of Trame et al. [24]. The results are shown as median with 25- and 75-quartile range (indicated as dotted lines). Diet groups that do not share indices (a–d) differ significantly (Kruskal–Wallis test followed by Benjamini–Hochberg correction (*p* < 0.05)).

Total serum zinc concentrations of omnivores, flexitarians, and vegetarians were comparable with 649.40 μg/L, 650.00 μg/L, and 647.10 μg/L, respectively. Vegans had the lowest serum zinc levels with a median of 604.10 μg/L. However, the difference was not significant compared to the other diet groups. Total serum zinc levels differed between men and women in the study collective, with men having slightly higher levels (*p* < 0.05). No differences in serum zinc levels were observed between men of the four groups, whereas the serum zinc concentrations of the female vegans were significantly lower compared to female omnivores and flexitarians (*p* = 0.02) (Table S1). The median serum zinc concentrations of all groups were located in the lower third of the reference range. Overall, the serum zinc concentrations of 22.5% omnivores, 21.3% flexitarians, 33.3% vegetarians, and 42.5% vegans fell below the reference range (Figure 2A).

The serum samples of omnivores, flexitarians and vegetarians contained 0.81, 0.79, and 0.79 nM free zinc, respectively, and did not differ between the groups. In contrast, the free zinc concentrations of vegans were significantly lower with 0.68 nM compared to the other dietary patterns (*p* ≤ 0.001) (Figure 2B). In women, the same group differences were observed as for the whole study collective (*p* ≤ 0.04), whereas in men, vegans had significantly lower free serum zinc levels in comparison to the flexitarians (*p* = 0.003) (Table S1). However, this sex-dependent difference might be caused by the smaller sample size of men in comparison to women. 

Omnivores had a median zinc intake of 11.99 mg/d, which differed significantly from the intakes of 9.61, 8.58, and 7.12 mg/d in flexitarians, vegetarians, and vegans (*p* ≤ 0.02), respectively. Furthermore, the lowest zinc intake in vegan subjects varied from the intake in flexitarians (*p* ≤ 0.01) (Figure 2C). Again, women had a lower zinc intake compared to men, with the same group differences being observed in women as for the whole study collective (*p* < 0.05). In men, omnivores also had the highest zinc intake, which varied from the lower intake in the other groups (*p* < 0.05) (Table S1).

The calculation of the Zinc and Phytate Diet Score revealed that the Phytate Diet Score increased gradually with the exclusion of animal foods from the diet as follows VN > VG > Flex > WD *(p* < 0.05) (Figure 2D). The Zinc Diet Score revealed comparable results as shown for the zinc intake as both were generated from the same dietary record (Table S1).

### 3.4. Baseline Copper Status 

**Figure 3:**
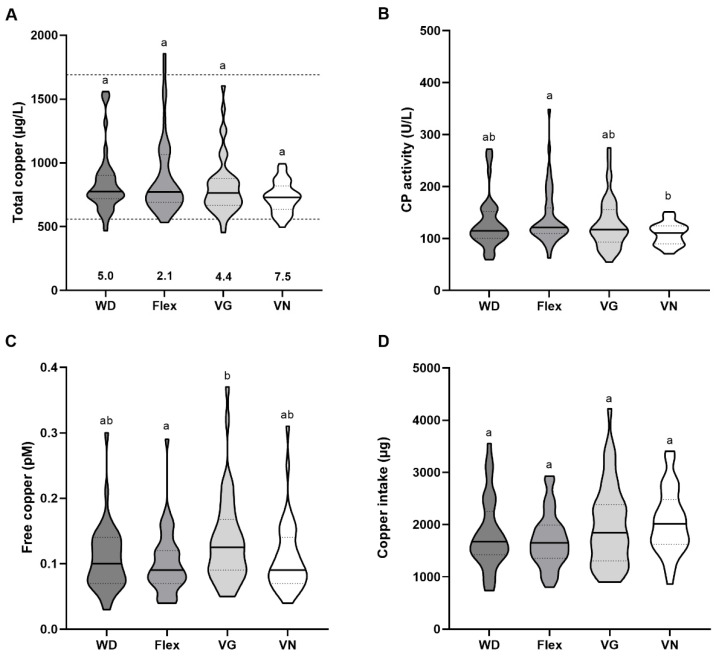
Analysis of copper biomarkers at baseline. (**A**) Total copper concentrations in the serum of omnivores (WD, *n* = 40), flexitarians (Flex, *n* = 47), vegetarians (VG, *n* = 45), and vegans (VN, *n* = 40) were determined using total reflection X-ray fluorescence spectroscopy (TXRF). The dashed lines indicate the reference range of copper in serum, with the numbers below the violin plots representing the percentage of subjects below this threshold. (**B**) Enzyme activity of Ceruloplasmin (CP) (WD, *n* = 38; Flex, *n* = 47; VG, *n* = 43; VN, *n* = 40) was measured by colorimetric assay. (**C**) Free serum copper levels were quantified by a fluorometric method using a copper-binding fluorescent peptide (FP4). (**D**) Daily copper intakes were calculated from baseline dietary records conducted over 5 days (WD *n* = 37, Flex *n* = 46, VG *n* = 39, and VN *n* = 38). The results are shown as median with 25- and 75-quartile range (indicated as dotted lines). Diet groups that do not share indices (a,b) differ significantly (Kruskal–Wallis test followed by Benjamini–Hochberg correction (*p* < 0.05)).

At baseline, no differences in serum copper concentrations were observed between the dietary patterns, and the median serum copper levels of all groups were within the reference range (Figure 3A). Serum copper levels differed between men and women in the study collective with generally higher values in women (*p* < 0.05). However, considering men and women separately did not reveal significant differences between the groups (Table S1). However, vegan women showed a trend towards lower serum copper concentrations compared to omnivores and flexitarians (*p* = 0.07). A small percentage of all groups had serum copper levels below the reference range of 560 μg/L with 5.0% of omnivores, 2.1% of flexitarians, 4.4% of vegetarians, and 7.5% of vegans (Figure 3A).

In accordance with total serum copper levels, the CP activity differed between men and women with generally higher values in women (Table S1). Again, the CP activity of omnivores, flexitarians, and vegetarians did not differ significantly. With a median of 110.80 U/L, vegans showed the lowest CP activity, which was significantly lower than the CP activity of flexitarians with a median value of 121.40 U/L (*p* = 0.02) (Figure 3B). 

The serum samples of omnivores, flexitarians, and vegans contained 0.10, 0.09, and 0.09 pM free copper. The vegetarian group showed the highest serum free copper levels with 0.13 pM, which was significantly higher compared to flexitarians (*p* = 0.03) (Figure 3C). In contrast to total serum copper, there was no sex-dependent difference in free serum copper (Table S1). 

The copper intake of the four groups did not differ significantly from each other (Figure 3D), even after considering men and women separately (Table S1).

### 3.5. Baseline Total Serum Copper/Zinc and Total Serum Selenium/Copper Ratios 

Besides considering individual serum trace element concentrations, the values were used to calculate the total copper to total zinc (Cu/Zn) ratio as well as the total selenium to total copper (Se/Cu) ratio as additional markers related to health. The Cu/Zn ratios as well as the Se/Cu ratios of the four groups did not differ significantly from each other. There was a sex-dependent difference in the Cu/Zn ratio, with women showing higher ratios (*p* < 0.05). Considering men and women separately did also not reveal significant differences between the groups (Table S1).

### 3.6. Serum Trace Element Concentrations during the Intervention

**Figure 4:**
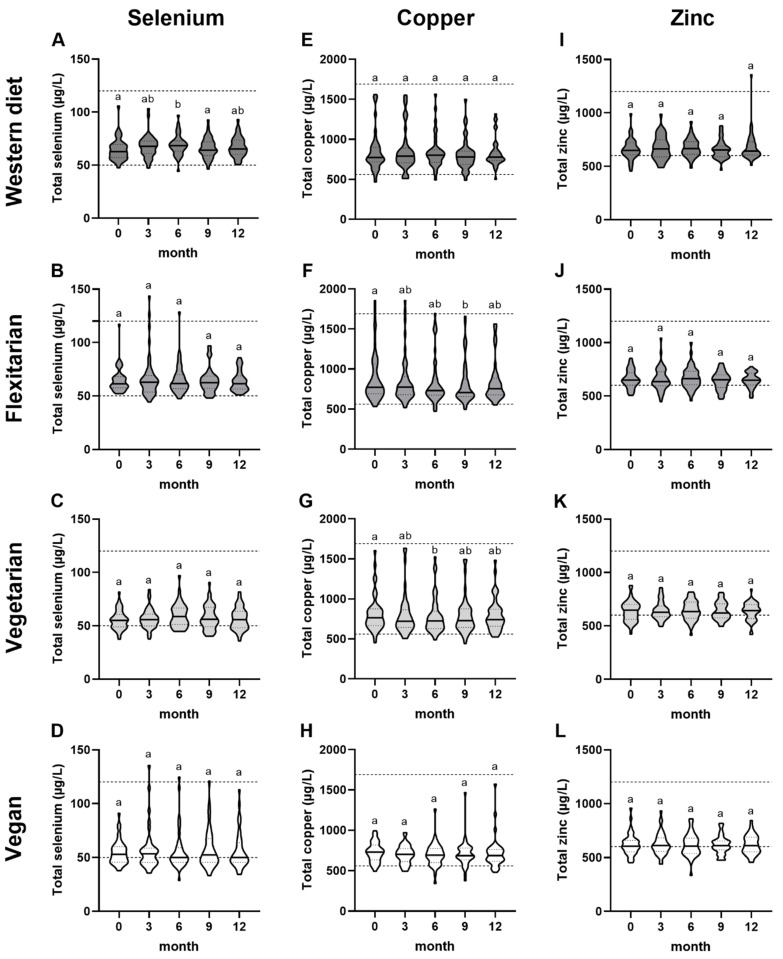
Comparison of trace element concentration during the study according to dietary pattern. (**A**–**D**) Total selenium, (**E**–**H**) total copper and (**I**–**L**) total serum zinc concentrations of omnivores (WD, *n* = 40), flexitarians (Flex, *n* = 47), vegetarians (VG, *n* = 45), and vegans (VN, *n* = 40) were determined using total reflection X-ray fluorescence spectroscopy (TXRF). The dashed lines indicate the reference ranges of serum trace element levels. The number 0 on the X-axis represents the baseline. The results are shown as median with 25- and 75-quartile range (indicated as dotted lines). Diet groups that do not share indices (a,b) differ significantly (Friedman test followed by Benjamini–Hochberg correction (*p* < 0.05)).

During the study, the group of omnivores showed a significant increase in serum selenium concentrations of 8% after 6 months compared to baseline (*p* = 0.003). However, serum selenium levels decreased significantly between month 6 and 9 (*p* = 0.003) and remained stable until the end of the study (Figure 4A). The serum selenium levels of the flexitarians, vegetarians and vegans did not change during the intervention (Figure 4B–D). After 3 months, serum selenium concentrations of two flexitarians and one vegan exceeded the reference range of 120 µg/L by 4%, 19%, and 13%, respectively. However, after 9 and 12 months, the levels were within the reference range again (Figure 4B,D). 

The median of total serum copper concentrations of flexitarians decreased by 9% after 9 months compared to baseline (*p* = 0.01) (Figure 4F), while in vegetarians a reduction of total serum copper levels of 5% was observed after 6 months (*p* = 0.03) (Figure 4G). In both vegetarian and flexitarian groups, the reduction in serum copper did not persist until the end of the study (12 months). In addition, considering men and women separately, neither flexitarians nor vegetarians showed a change in serum copper concentrations over the course of the study (Table S2). No change in serum copper concentration was observed within the study in the omnivorous and vegan groups (Figure 4E,H). 

Regarding Se/Cu ratios, a significant increase was evident in omnivores (8–10%) as well as vegetarians (2–4%) after 6 (*p* ≤ 0.02) and 9 months (*p* ≤ 0.03) compared to baseline. However, the total Se/Cu ratios for both groups were not significantly different anymore after 12 months compared to the baseline (Table S2). The total serum zinc concentrations (Figure 4I–L) as well as Cu/Zn ratios, GPX and CP activity in the serum of the participants remained unchanged in all groups over the entire study period (Table S2). In addition to the time course, the percentage changes of the observed parameters from baseline to 12 months were compared for the individual groups. There were no differences between the four groups for any of the above-described parameters (Table S2). 

Overall, the observed baseline group differences in total serum selenium concentration and GPX activity were maintained over the course of the study. In addition, the baseline group differences in serum zinc levels of the vegan women compared to omnivores and flexitarians were also evident after 6 and 12 months. Regarding copper status, a lower total serum copper concentration and CP activity was observed in vegans compared to omnivores and flexitarians from 3 months until the end of the intervention period (*p* < 0.05). This difference was observed when considering all study participants but also for the subgroup of women (Table S2). 

### 3.7. Correlation Analysis of the Investigated Parameters 

**Figure 5:**
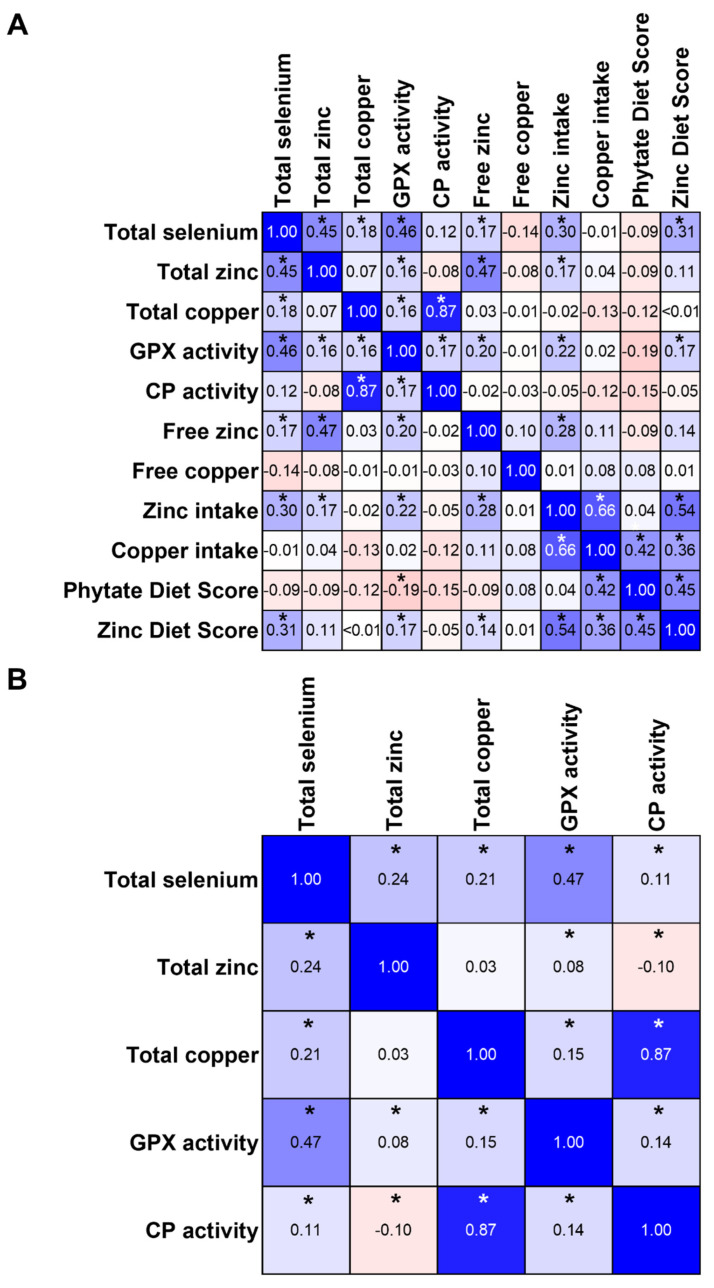
Correlation analysis of the investigated parameters at (**A**) baseline and (**B**) during the remaining study period (3, 6, 9, 12 months). The Squares indicate Spearman’s correlation coefficients. Squares marked with an asterisk indicate statistical significance (*p* < 0.05).

At baseline, total serum selenium showed a positive correlation with GPX activity (*p* < 0.001). Furthermore, the concentrations of total serum copper correlated strongly and significantly with serum CP concentrations (*p* < 0.001) as expected. Interestingly, the copper intake correlated with the Phytate Diet Score and Zinc Diet Score (*p* < 0.001). A moderate correlation was observed between the total and free zinc concentrations (*p* < 0.001) as well as zinc intake and Zinc Diet Score (*p* < 0.001). Interestingly, total serum selenium correlated moderately with total serum zinc, zinc intake, and the Zinc Diet Score (*p* < 0.001). Less stringent correlation was observed between total serum selenium and total serum copper (*p* < 0.001) as well as free serum zinc (*p* = 0.024). In addition, GPX activity showed a weak correlation with total serum zinc (*p* < 0.001), copper (*p* < 0.001), CP activity (*p* < 0.001), free zinc (*p* = 0.01), zinc intake (*p* < 0.001) and the Zinc Diet Score (*p* < 0.03). Moreover, a weak correlation was observed between zinc intake and total as well as free serum zinc (*p* < 0.03). The level of free copper in serum showed no correlation with any of the other investigated parameters (Figure 5A). 

The correlations between total serum selenium and GPX activity (*p* < 0.001) and total copper and CP activity (*p* < 0.001) remained throughout the study period. In addition, all investigated parameters showed weak correlations with each other over the course of the study (*p* < 0.05), except for total serum copper and zinc, which did not correlate (Figure 5B).

## 4. Discussion

The data on trace element supply showed substantial differences between meat-containing diets and vegetarian/vegan diets that were most pronounced for selenium. Both selenium biomarkers were significantly lower in vegetarians and vegans (Figure 1A). Moreover, more vegetarians and vegans had selenium concentrations below the reference range, indicating that these study participants have a higher risk of developing health impairments such as colorectal, liver, or breast cancer due to their low selenium status [34]. Our results are in line with other studies showing that individuals with predominantly or purely plant-based diets have a lower selenium status measured by serum/plasma selenium concentration [35,36,37,38,39], GPX activity [35,37], and/or SELENOP levels [39,40]. In this study, we decided against adding SELENOP as selenium biomarker because GPX activity already showed group differences (Figure 1B) as expected from the selenium concentrations and a correlation between serum GPX activity and total selenium was observed (Figure 5A,B). GPX activity usually becomes saturated above 80–90 µg/L [41,42,43], while SELENOP covers selenium concentration ranges up to 120 µg/L [41]. As most of the study participants had baseline selenium concentrations below 80 µg/L (except for 6%) it was not necessary to add the additional biomarker. In line with this, a separate calculation considering only participants with serum selenium concentrations below 80 μg/L showed that the correlation between GPX activity and selenium concentration was further increased over the entire study period (Figure S1). 

The overall selenium status of the omnivores in NuEva is in accordance with published data, e.g., from the European Prospective Investigation into Cancer and Nutrition (EPIC) with average serum selenium concentrations of the population in Central Europe and Germany of 78.20 μg/L and 73.75 μg/L, respectively [34]. From a public health perspective, it is most critical that the percentage of participants with a serum selenium concentration below 50 µg/L accounts for 33–40% in vegetarians and vegans which is rarely observed in the meat-eating groups (Figure 1A). Thus, those vegetarians and vegans would profit from an improved selenium supply. In addition, data from the EPIC-Oxford follow-up, which investigated the nutrient intake of a representative sample of 6403 vegetarians and 761 vegans, showed that 43–60% of vegetarians and 33–49% of vegans did not reach the recommended intake for selenium [21]. In contrast to the data shown here, there are also studies reporting no effect of plant-based diets on the selenium status. For example, the serum selenium concentrations of healthy German vegans (68 µg/L) aged 30–57 years did not differ from omnivores (77 µg/L) and no values below the reference range were reported [40]. Most probably, the inconsistency of the results is caused by regional differences of selenium concentration of plant-based food items and/or the frequency of the consumption of imported food items from regions with higher soil selenium concentrations. Overall, selenium is one of the potentially critical trace elements when consuming a plant-based diet.

The zinc status is usually assessed by analyzing serum zinc concentrations [44] even though it is known that changes in this parameter are only detectable in individuals with zinc deficiency and not with a suboptimal zinc status. Based on present knowledge, serum zinc in combination with data about the nutrition-based zinc supply is supposed to be the best indicator of zinc status [45]. In contrast to many other studies from Europe showing lower serum/plasma zinc levels of 780–810 µg/L in vegetarians compared to 850–910 µg/L in omnivores [35,46], we found no overall differences in the serum zinc concentration (Figure 2A). A meta-analysis revealed that the serum zinc concentrations of vegetarians and vegans were 5.9 μg/L and 7.7 μg/L lower than those of omnivores, respectively [19]. In addition, a larger percentage of vegetarians (19–24%) and vegans (47–50%) had serum zinc concentrations below the reference range compared to omnivores (11–18%) [46,47], which was also observed in the NuEva study (Figure 2A). Considering vegans, again previous studies on healthy subjects aged 18–57 years have consistently shown that vegans have significantly lower serum/plasma zinc concentrations than omnivores [40,46,47]. However, no sex-specific differences were reported in these studies, but in the NuEva study, a significantly lower serum zinc level was only observed for female vegans (Table S1). In addition, vegan women had the lowest baseline zinc intake compared to omnivores and flexitarians (Table S1) and lower urinary zinc excretion was observed in vegetarians and vegans compared to omnivorous subjects in the NuEva study [14]. As zinc homeostasis is tightly regulated, it is possible that a lower zinc intake was compensated by decreased excretion and/or increased absorption which provides an explanation for rather stable serum zinc concentrations. 

In serum, zinc is mainly bound to transport proteins such as albumin and α-macroglobulin [48], but a subnanomolar fraction of free zinc is detectable in serum as well. This fraction is also known as available and biologically active zinc pool [23]. Changes in free zinc concentrations are considered to be a suitable biomarker for alterations in zinc homeostasis [22]. The concentrations of free zinc in the NuEva collective are comparable to the healthy control subjects (0.8 ± 0.3 nM) reported by Maares et al. who investigated changes in free zinc concentrations in subjects diagnosed with COVID-19 [49]. In the NuEva study, women had lower free zinc concentrations compared to men (Figure 2B), which was also observed in a previous study [23]. A moderate correlation with total zinc was observed (Figure 5A), which is also in line with the literature [49]. In accordance with total serum zinc, vegan women showed the lowest free zinc concentrations compared to the other groups (Figure 2B). 

Due to the lack of alternative biomarkers, the Zinc Diet Score was developed to better predict an individual’s risk of zinc deficiency [24]. In addition to calculating the zinc intake from the dietary records at baseline, the Zinc and the Phytate Diet Scores were used to more specifically assess food items that affect the zinc intake as well as their portion size and frequency of consumption. The Zinc Diet Score of vegans was lower compared to omnivores (Table S1), which is in line with the reduction of serum zinc in vegan women. In contrast, the Phytate Diet Score increased gradually with the exclusion of animal foods (VN > VG > Flex > WD; *p* < 0.05) (Figure 2D). This fits to the concept of lower bioavailability of zinc from a plant-based diet, as especially phytic acid forms insoluble chelate complexes with divalent cations such as zinc, resulting in decreased absorption of the trace element [50]. Accordingly, the revised D-A-CH reference values for zinc intake were adjusted to a phytate intake in the range of 300–900 mg/day [51]. It is currently not possible to reliably determine the phytate intake, as no information on the phytate content of foods is available in databases. The Phytate Diet Score could, therefore, approximate the phytate intake and thus provide an estimate of the bioavailability of zinc from food. The high Phytate Diet Scores of vegetarians and vegans in the NuEva study (Figure 2D) indicate that women and men who eat a purely or predominantly plant-based diet should achieve a zinc intake of at least 10 mg/day for women and 16 mg/day for men. However, we showed that the zinc intake of vegetarian and vegan NuEva participants is clearly below this recommended daily intake, at 10.7 and 9.7 mg/day for men and 8.1 and 6.8 mg/day for women, respectively (Table S1). This was also the result of a systematic review, which showed that the zinc intake of vegetarians and vegans fell below the recommended daily intake when the lower bioavailability from plant-based diets was considered [52].

Regarding the copper status, no differences were observed at baseline for serum copper concentration, while the CP activity of vegans was lower compared to flexitarians (Figure 3A,B). Furthermore, a trend towards lower copper concentrations in female vegans compared to omnivores and flexitarians emerged (Table S1). Similar to the NuEva study, a Slovakian study in healthy subjects aged 34–60 years revealed no differences in plasma copper concentrations in vegetarians and vegetarian women compared to omnivorous subjects [53]. In contrast, there is evidence that the total copper concentration of healthy Slovakian vegetarians aged 20–57 is lower compared to omnivorous subjects [35]. This has been confirmed in a cohort of postmenopausal vegetarian women who had been following their diet for 20 years, although their copper intake was higher than that of omnivorous women (1.55 vs. 1.33 mg/d) [54]. In one study, it was observed that switching to a vegetarian diet resulted in a decrease in plasma copper concentration [55]. Both a higher copper intake but also a reduced copper bioavailability are possible when consuming a predominantly or purely plant-based diet. Here we found no differences between the copper intake of the groups (Figure 3D) and a strong correlation between copper intake and the Phytate Diet Score (Figure 5A). However, the copper status is generally strongly regulated and this regulation mainly takes place in the liver where excess copper can be efficiently excreted via the bile. In addition, copper deficiency as well as copper excess are very rarely observed in Europe indicating that small changes in copper intake and/or bioavailability are of minor relevance [56]. In the NuEva study, the copper intake was above the recommended reference value of 1000–1500 mg for all groups and only a small percentage of study participants had serum copper concentrations that were outside the reference range (Figure 3A). 

One of the major reasons to include the analysis of copper status in the present study was the calculation of Cu/Zn and Se/Cu ratios, which are well established biomarkers for inflammatory and infectious diseases [57,58,59]. Trace elements can interact with each other and thus, ratios of trace elements are considered as a suitable biomarker to detect early shifts in trace element homeostasis [22]. Although the groups differed regarding the individual trace element supply, no differences in trace element ratios between the groups were observed (Table S1). One study compared the Cu/Zn ratio of omnivores and vegetarians and also showed no differences between the groups [35]. Overall, a predominantly or purely plant-based diet does not seem to cause shifts in trace element ratios. 

In addition to the observed differences at baseline, the NuEva study provides the exclusive opportunity to follow the trace element supply and status over one year. The intervention through standardized menu plans was intended to not only monitor the subjects’ diet but also to optimize their intake of various nutrients. However, this attempt was limited for selenium because databases of foods do not contain information about their selenium content. Overall, the observed differences in selenium concentration, GPX activity, and total zinc in women between the dietary patterns were maintained, indicating that the menu plans were not suitable to adjust the selenium and zinc intake in the groups with plant-based diets to the level of omnivores (Table S2). Only in the group of omnivores, the serum selenium concentration changed significantly over the course of the study. It increased after 6 months compared to the beginning of the study but dropped back to baseline levels after 9 months (Figure 4A) This increase affected the Se/Cu ratio, which also decreased again after 9 months (Table S2). It is possible that the foods provided in the menu plans during the first few months provided more selenium than later in the study, or that the daily intake of meat and sausage products contributed significantly to selenium intake. However, since the selenium intake of the subjects is unknown, due to the lack of information on the selenium content of foods in the underlying database, this cannot be conclusively clarified. In the MoKaRi study, the selenium status of subjects with a high risk of cardiovascular disease was modulated by a nutritional intervention with optimized daily menu plans [60]. The characteristics of the daily menu plans were comparable to those of the NuEva study and aimed to achieve a flexitarian diet. In contrast to the NuEva study, serum selenium decreased in participants with initially adequate serum selenium concentrations (>80 µg/L) after 20 weeks of intervention. Similarly, the selenium intake could not be adjusted due to the lack of information on selenium content [60], which in general also limits the possibility of systematically improving selenium intake through diet. 

In addition, the optimized menu plans were characterized by a fiber intake of >40 mg/d. We expected an inverse relationship between increased dietary fiber intake and serum zinc levels at least in omnivores and flexitarians, which had baseline fiber intakes of 24.4 and 27.0 g/d [14], respectively, as it has already been shown that an increase in dietary fiber intake simultaneously increases phytate intake and thus the risk of suboptimal zinc supply [61]. Furthermore, in the NuEva study the Phytate Diet Score correlated with the daily fiber intake (Figure S2). In addition, a change from an omnivorous to a vegetarian diet in healthy subjects led to a 13% reduction in serum zinc concentration after three months, which was explained by a higher dietary fiber (44 g) and thus phytate intake [55]. Nevertheless, in the NuEva study, the increase in dietary fiber (omnivores and flexitarians) or the consistently high intake (vegetarians and vegans) had no effect on serum zinc levels over the course of the study (Table S2). This was also observed in a collective of men with elevated cholesterol levels, in which no association between a dietary intervention with increased fiber intake (30 g/d) and plasma zinc level was apparent, even with higher dietary fiber amounts (25 g/1000 kcal) [62]. This illustrates that dietary fiber intake does not necessarily lead to a reduction in serum zinc levels if the zinc intake remains stable. It further needs to be taken into account that foods high in fiber such as wholegrains and nuts also serve as plant-based sources of zinc [63]. In addition, it must be considered that similar to copper, zinc homeostasis is tightly regulated over a wide range of dietary intakes by adjusting absorption and/or excretion [64]. Besides the tight homeostatic regulation of trace elements, non-dietary factors such as infections, inflammations, hormonal status, ageing, and the use of medications influence the concentrations of trace elements as well [22]. 

Overall, the use of optimized menu plans could not evidently compensate the observed differences between the groups at baseline. Finally, it must be considered that results from human trials show a high individual variability and can be influenced by external factors as the results depend on the individual compliance of the subjects. Over a period of 12 months, it was not possible to fully assess compliance, which is a factor of uncertainty.

## 5. Conclusions

The NuEva study enables the comparison of four dietary patterns (omnivores, flexitarians, vegetarians, and vegans) with respect to their effects on the status of the trace elements selenium, zinc, and copper over a period of 12 months. Overall, biomarkers of selenium and zinc were lower in vegetarians and especially vegans, confirming that a sufficient supply of these trace elements is more difficult to achieve when following a plant-based diet. The observed group differences at baseline, especially for selenium but also zinc (females), were evident throughout the study and thus strengthen the characterization of the dietary patterns. The investigated trace element status biomarkers remain relatively stable during the study in the investigated diet groups and, therefore, the intervention using optimized menu plans did not lead to a long-term change in trace element supply. The results highlight the underlying problem that missing or imprecise information on the trace element content of foods still complicates a reliable representation of trace element intake and does not permit to optimize trace element status via menu plans. Thus, specific recommendations for an improvement of dietary trace element supply can only be given to a limited extent. Nuts (especially Brazil nuts in case of selenium), seeds, wheat bran, and barley flakes might be suitable food sources of zinc and selenium and should be consumed regularly in vegetarian and vegan diets. In addition, strategies to reduce phytate content, such as soaking, fermenting, or sprouting, should be increasingly recommended to vegans and vegetarians.

## Figures and Tables

**Table 1:** Baseline characteristics of the NuEva collective (*n* = 172, median/interquartile range (IQR); (min–max)).

	Sex	WD 13 m, 27 w		Flex 9 m, 38 w		VG 13 m, 32 w		VN 12 m, 28 w	
Age (years)	all	32.0/17.0 (20.0–56.0)	a	28.0/21.0 (19.0–69.0)	a,b	28.0/19.0 (18.0–65.0)	a,b	25.0/10.0 (19.0–56.0)	b
Weight (kg)	all	74.0/29.1 (49.5–124.6)	a	62.6/9.1 (51.1–87.6)	b	67.0/16.3 (50.0–94.6)	a,b	60.9/17.0 (47.6–84.4)	b
	w	68.7/18.9 (49.5–100.9)	a	59.7/9.9 (51.1–87.2)	b	63.3/13.6 (50.0–92.5)	a,b	57.4/10.0 (47.6–80.0)	b
	m	79.9/17.45 (68.0–124.6)	a	68.7/12.6 (62.6–87.6)	a	80.7/17.5 (66.6–94.6)	a	71.6/8.1 (50.3–84.4)	a
BMI (kg/m^2^)	all	24.0/5.0 (19.0–40.0)	a	22.0/3.0 (18.0–31.0)	b	23/3.5 (19.0–30.0)	a,b	22/3.0 (17.0–27.0)	b

WD = omnivores, Flex = flexitarians, VG = vegetarians, VN = vegans, w = women, m = men. Diet groups that do not share indices differ significantly (*p* < 0.05). The letter describes the group size.

## Data Availability

The datasets generated and analyzed during the current study are available from the corresponding authors on reasonable request. The data are not publicly available due to the ongoing evaluation of the data sets by the study team.

## Supplementary Materials

The following supporting information can be downloaded at: https://www.mdpi.com/article/10.3390/nu15163538/s1, Figure S1: Correlation analysis of total serum selenium and GPX activity over the entire study period; Figure S2: Correlation analysis of total fiber intake and Phytate Diet Score; Table S1: Baseline trace element status of the study collective—NuEva-screening; Table S2: Total trace element concentrations and biomarkers in serum of participants over the course of the Study.
