# Supplementary material for: Selenium, Zinc, and Copper Status of Vegetarians and Vegans in Comparison to Omnivores in the Nutritional Evaluation (NuEva) Study
Source: Nutrients. 2023 Aug 11;15(16):3538. doi: 10.3390/nu15163538 (PMC10459941; doi:10.3390/nu15163538)
Supplement: Supplementary file 1 [file nutrients-15-03538-s001.zip › nutrients-2530221-supplementary.pdf]

Supplementary Material

Table S1: Baseline trace element status of the study collective - NuEva-screening.

		WD		Flex		VG		VN	
**Total selenium** (µg/L)	all	62.85 / 12.44	a	61.37 / 10.44	a	55.12 / 11.77	b	52.84 / 14.26	b
**Total zinc** (µg/L)	all	649.40 / 113.40	a	650.00 / 115.70	a	647.10 / 138.70	a	604.10 / 106.50	a
	w	651.50 / 74.50	a	644.60 / 100.70	a	611.10 / 159.40	a,b	592.40 / 107.30	b
	m	668.70 ± 98.81	a	721.60 ± 85.54	a	647.70 ± 61.45	a	683.80 ± 89.25	a
**Free zinc** (nM)	all	0.81 / 0.27	a	0.79 / 0.29	a	0.79 / 0.17	a	0.68 / 0.12	b
	w	0.80 ± 0.15	a	0.76 ± 0.18	a	0.80 ± 0.18	a	0.68 ± 0.13	b
	m	0.88 / 0.31	a,b	0.92 / 0.33	a	0.79 / 0.23	a,b	0.69 / 0.14	b
**Zinc intake** (mg/d)	all	11.99 / 5.05	a	9.61 / 4.56	b	8.58 / 3.55	b,c	7.12 / 3.39	c
	w	10.99 / 3.97	a	9.08 / 4.40	b	8.07 / 3.14	b,c	6.80 / 2.34	c
	m	15.99 ± 5.57	a	11.65 ± 1,47	b	10.74 ± 4.48	b	9.71 ± 2.88	b
**Total copper** (µg/L)	all	774.20 / 182.50	a	772.60 / 375.00	a	764.10 / 208.80	a	729.20 / 182.10	a
	w	835.50 / 189.70	a	795.10 / 378.70	a	703.1 / 216.80	a	754.30 / 209.40	a
	m	697.70 ± 128.50	a	677.60 ± 98.89	a	691.70 ± 120.40	a	639.50 ± 82.88	a
**Free copper** (pM)	all	0.10 /0.07	a,b	0.09 /0.05	a	0.13 /0.08	b	0.09 /0.07	a,b
**Copper intake** (µg/d)	all	1672 / 626	a	1649/ 632	a	1842 / 1080	a	2014 / 856	a
	w	1582 ± 552	a	1657 ± 529	a	1769 ± 628	a	1917 ± 584	a
	m	2261 ± 716	a	1883 ± 465	a	2512 ± 881	a	2370 ± 591	a
**Cu/Zn**	all	1.23 / 0.50	a	1.16 / 0.50	a	1.23 / 0.45	a	1.18 / 0.37	a
	w	1.35 / 0.49	a	1.30 / 0.54	a	1.27 / 0.47	a	1.26 / 0.31	a
	m	1.07 / 0.26	a	0.95 / 0.13	a	1.08 / ± 0.24	a	1.00 / ± 0.14	a
**Se/Cu**	all	0.08 / 0.02	a	0.08 / 0.03	a	0.07 / 0.02	a	0.07 / 0.02	a
**GPX activity** (U/L)	all	285.90 / 74.20	a	281.00 / 66.60	a	253.1 / 67.80	b	238.40 / 79.90	b
**CP activity** (U/L)	all	114.80 / 51.60	a	121.40 / 49.50	a	117.2 / 62.40	a	110.80 / 34.59	a
	w	125.10 / 51.10	a	125.10 / 65.50	a	123.70 / 66.40	a	117.00 / 26.10	a
	m	98.66 ± 27.90	a	103.00 ± 23.05	a	100.10 ± 23.88	a	95.75 ± 11.43	a
**Zinc Diet Score**	all	141.80 / 57.60	a	124.50 / 53.20	a,b	124.60 / 67.7	a,b	123.70 / 64.50	b
	w	135.90 / 30.10	a	123.00 / 47.90	a	119.60 / 69.40	a	121.90 / 50.62	a

	m	185.70 ± 36.64	a	156.40 ± 49.9	a	138.50 / 69.64.	a	148.1 / 56.88	a
**Phytate Diet Score**	all	20750 / 18125	a	26200 / 24040	b	41060 / 35899	c	59765 / 36642	d

WD = omnivores, Flex = flexitarians, VG = vegetarians, VN = vegans, w = women, m = men, GPX = glutathione peroxidase, CP = ceruloplasmin, Cu/Zn= copper/zinc ratio, Se/Cu = selenium/copper ratio

Results are presented as mean ± SD or Median / IQR, Diet groups that do not share indices differ significantly (ordinary one-way ANOVA or Kruskall-Wallis test followed by Benjamini-Hochberg correction ($p < 0.05$))

Table S2: Total trace element concentration and biomarkers in serum of participants over the course of the study.

	Sex	t	WD	p†	◊*	Flex	p†	◊*	VG	p†	◊*	VN	p†	◊*
**Total selenium** (µg/L)	all	0	62.85 / 12.44	a	a	61.37 / 10.44	a	a	55.12 / 11.77	a	b	52.84 / 14.26	a	b
		3	67.75 / 11.71	a,b	a	62.78 / 15.59	a	a	55.85 / 11.11	a	b	53.34 / 13.16	a	b
		6	68.42 / 9.77	b	a	61.51 / 12.99	a	b	58.67 / 15.44	a	c	50.03 /13.28	a	d
		9	64.24 / 12.84	a	a	62.42 / 11.48	a	a	56.03/18.17	a	b	52.33 / 22.04	a	b
		12	65.33 / 13.56	a,b	a	61.43 / 12.24	a	a	55.78 / 15.44	a	b	50.06 / 18.12	a	b
		%	4.35 / 24.58		a	-2.50 / 15.76		a	1.32 / 24.36		a	-2.62 / 27.88		a
**GPX activity** (U/L)	all	0	285.90 / 74.20	a	a	281.00 / 66.60	a	a	253.10 / 67.80<br>251.40 ± 56.50	a	b	238.40 / 79.90	a	b
		3	298.90 / 71.80	a	a	281.70 / 72.00	a	a	254.80 / 59.10<br>247.40 ± 39.01	a	b	221.60 / 44.30	a	c
		6	286.80 / 81.50<br>289.60 ± 50.59	a	a	297.10 / 66.70<br>289.30 ± 55.10	a	a	266.80 / 68.20<br>262.60 ± 40.39	a	b	230.20 / 53.60<br>231.00 ± 42.41	a	c
		9	285.00 / 88.20<br>286.40 ± 53.67	a	a	285.00 / 47.40<br>277.00 ± 49.57	a	a,b	261.90 / 58.60<br>260.70 ± 45.66	a	a,b	231.20 / 62.30<br>237.20 ± 48.37	a	c
		12	285.50 / 78.90	a	a	272.10 / 50.50	a	a	258.20 / 66.10<br>255.80 ± 41.28	a	b	232.60 / 71.70	a	c
		%	-0.25 / 14.43		a	-0.66 / 19.74		a	-1.50 / 22.83		a	-3.32 / 24.52		a
**Total zinc** (µg/L)	all	0	649.40 / 113.40	a	a	650.00 / 115.70	a	a	647.10 / 138.70	a	a	604.10 / 106.50	a	a
		3	662.90 / 159.70	a	a	634.60 / 130.30	a	a	628.30 / 100.30	a	a	610.10 / 126.30	a	a
		6	665.50 / 117.70	a	a	662.70 / 127.30	a	a	634.00 / 151.60	a	a	605.90 / 143.40	a	a
		9	652.60 / 100.40	a	a	651.50 / 118.30	a	a	620.30 / 131.90	a	a	609.50 / 83.00	a	a
		12	642.60 / 124.40	a	a	647.80 / 110.50	a	a	643.50 / 130.30	a	a	609.30 / 136.30		a
		%	3.28 / 16.58		a	-0.48 / 18.23		a	3.39 / 22.42		a	0.06 / 18.76		a
	w	0	651.50 / 74.50	a	a	644.60 / 100.70	a	a	611.10 / 159.40<br>632.10 ± 107.60	a	a,b	592.40 / 107.30	a	b
		3	615.80 / 134.80	a	a	625.20 / 88.00	a	a	632.10 / 101.40<br>647.00 ± 95.41	a	a	588.60 / 111.10	a	a
		6	652.40 / 90.90<br>653.40 ± 71.17	a	a	646.70 / 112.90<br>655.50 ± 87.80	a	a	643.60 ± 96.44	a	a	585.80 / 116.00<br>591.20 ± 99.18	a	b
		9	625.50 / 87.80<br>633.40 ± 74.51	a	a	638.90 / 122.80<br>627.20 ± 80.26	a	a	639.90 ± 82.23	a	a	598.70 / 108.40<br>597.50 ± 72.97	a	a
		12	632.40 / 100.80	a	a	642.90 / 88.40	a	a	639.10 / 126.30<br>625.20 ± 86.63	a	a,b	588.30 / 106.00	a	b
		%	1.72 / 16.93		a	0.25 / 18.89		a	-0.42 / 24.75		a	2.14 / 17.75		a
	m	0	668.70 ± 98.81	a	a	721.60 ± 85.54	a	a	647.70 ± 61.45	a	a	683.80 ± 89.25	a	a
		3	733.30 ± 94.02	a	a	737.50 ± 82.98	a	a	733.30 ± 94.02	a	b	671.70 ± 100.40	a	a,b
		6	720.70 ± 99.87	a	a	722.50 ± 139.60	a	a	720.70 ± 99.87	a	a	681.70 ± 98.83	a	a
		9	707.80 ± 92.69	a	a	706.40 ± 72.40	a	a	707.80 ± 92.69	a	a	661.50 ± 97.16	a	a

		12	712.60 ± 95.33	a	a	674.40 ± 64.72	a	a	712.60 ± 95.33	a	a	672.20 / 113.30	a	a
		%	7.61 ± 14.34		a	-5.77 / 11.16		a	7.61 / 14.34		a	-0.78 / 18.80		a
**Total copper** (µg/L)	all	0	774.20 / 182.50	a	a	772.60 / 375.00	a	a	764.10 / 208.80	a	a	729.20 / 182.10	a	a
		3	790.50 / 184.30	a	a	774.40 / 227.00	a,b	a	720.70 / 218.90	a,b	a,b	700.40 / 157.80	a	b
		6	802.20 / 158.50	a	a	730.80 / 194.80	a,b	a,b	726.10 / 218.70	b	a,b	693.30 / 171.40	a	b
		9	778.40 / 166.40	a	a	706.70 / 219.10	b	a,b	728.60 / 231.40	a,b	a,b	685.90 / 143.60	a	b
		12	776.70 / 120.00	a	a	754.50 / 206.90	a	a	742.80 / 212.00	a,b	a	687.60 / 144.30	a	b
		%	0.89 / 20.61		a	-4.39 / 14.19		a	-1.26 / 18.08		a	-2.73 / 19.92		a
	w	0	835.50 / 189.70	a	a	795.10 / 378.70	a	a	804.2. / 216.80	a	a	754.30 / 209.40	a	a
		3	820.40 / 390.50	a	a	784.30 / 299.60	a	a,b	779.30 / 250.60	a	a,b	730.30 / 124.80	a	b
		6	822.20 / 189.80	a	a	759.90 / 239.60	a	a,b	760.10 / 281.70	a	a,b	695.90 / 135.40	a	b
		9	836.50 / 187.00	a	a	756.70 / 268.50	a	a,b	778.40 / 247.50	a	a,b	693.70 / 118.30	a	b
		12	800.10 / 188.20	a	a	791.50 / 244.60	a	a	792.6 / 241.60	a	a	717.20 / 142.20	a	b
		%	0.00 / 22.00		a	-3.50 / 14.25		a	-2.00 / 20.70		a	-3.00 / 24.75		a
	m	0	718.30 / 122.20<br>697.70 ± 128.50	a	a	662.70 / 171.80<br>677.60 ± 98.89	a	a	697.70 ± 128.50	a	a	680.50 ± 88.33	a	a
		3	713.80 / 130.60<br>715.10 ± 110.90	a	a	655.10 / 169.50<br>683.50 ± 111.50	a	a	715.10 ± 115.50.	a	a	657.30 ± 111.00	a	a
		6	709.40 / 160.00<br>727.40 ± 115.50	a	a	639.30 / 167.50<br>628.50 ± 108.10	a	a	727.40 ± 115.50	a	a	662.20 ± 112.50	a	a
		9	692.70 / 122.80	a	a	629.90 / 98.80	a	a	662.20 / 154.40<br>686.00 ± 87.40	a	a	627.70 / 186.30<br>633.30 ± 119.60	a	a
		12	732.80 / 97.80	a	a	675.60 / 153.90	a	a	694.30 / 152.20<br>728.10 ± 85.12	a	a	628.70 / 151.70<br>639.50 ± 82.88	a	a
		%	2.00 / 18.00		a	-5.00 / 16.00		a	2.00 / 18.00		a	-4.00 / 18.50		a
**CP activity (U/L)**	all	0	114.80 / 51.60	a	a,b	121.40/ 49.50	a	a	117.20 / 62.40	a	a,b	110.80 / 34.59	a	b
		3	121.20 / 47.00	a	a	122.50 / 61.26	a	a	115.7 / 33.52	a	a,b	103.30 / 37.36	a	b
		6	120.50 / 25.50	a	a	115.80 / 40.47	a	a	112.60 / 43.98	a	a,b	101.70 / 37.29	a	b
		9	117.30 / 49.47	a	a	115.10 / 40.57	a	a	112.10 / 48.55	a	a	97.33 / 36.55	a	b
		12	113.60 / 29.60	a	a	118.90 / 25.40	a	a	116.70 / 59.24	a	a	96.83 / 28.72	a	a
		%	1.27 / 28.94		a	-2.87 / 22.73		a	-0.37 / 28.60		a,b	-6.27 / 33.41		b
	w	0	125.10 / 51.60	a	a	125.10 / 65.50	a	a	123.70 / 66.40	a	a	117.00 / 26.10	a	a
		3	128.90 / 50.00	a	a	127.50 / 62.50	a	a	125.60 / 30.30	a	a,b	105.20 / 41.92	a	b
		6	127.40 / 32.00	a	a	120.90 / 43.50	a	a,b	124.00 / 53.90	a	a,b	104.70 / 34.83	a	b
		9	126.00 / 35.20	a	a	120.00 / 45.75	a	a	137.70 / 65.00	a	a	103.60 / 34.88	a	b

		12	123.50 / 45.10	a	a	120.80 / 58.80	a	a	125.00 / 77.93	a	a,b	109.40 / 32.07	a	b
		%	-2.17 / 35.21		a	-2.36 / 19.64		a	-1.55 / 21.17		a	-3.85 / 38.51		a
	m	0	98.66 ± 27.90	a	a	103.00 ± 23.05	a	a	100.10 ± 23.88	a	a	95.75 ± 11.43	a	a
		3	106.80 ± 27.48	a	a	98.95 ± 23.55	a	a	94.11 ± 15.47	a	a	90.33 ± 23.46	a	a
		6	99.12 ± 25.42	a	a	94.41 ± 20.34	a	a	92.58 ± 16.83	a	a	93.61 ± 16.32	a	a
		9	94.63 ± 13.84	a	a	95.15 ± 25.15	a	a	94.20 ± 16.18	a	a	92.01 ± 19.18	a	a
		12	101.90 ± 14.33	a	a	100.90 ± 22.64	a	a	97.84 ± 23.81	a	a	85.88 ± 11.55	a	a
		%	8.51 ± 24.27		a	0.00 ± 20.78		a	-0.48 ± 17.87		a	-9.60 ± 13.74		a
**Cu/Zn**	all	0	1.23 / 0.50	a	a	1.16 / 0.50	a	a	1.23/ 0.45	a	a	1.18 / 0.37	a	a
		3	1.19 / 0.38	a	a	1.25 / 0.42	a	a	1.18 / 0.39	a	a	1.08 / 0.28	a	a
		6	1.17 / 0.33	a	a	1.16 / 0.54	a	a	1.12 / 0.44	a	a	1.12 / 0.32	a	a
		9	1.19 / 0.45	a	a	1.14 / 0.42	a	a	1.13 / 0.45	a	a	1.16 / 0.31	a	a
		12	1.23 / 0.31	a	a	1.22 / 0.38	a	a	1.24 / 0.45	a	a	1.13 / 0.30	a	a
		%	-0.46 / 12.73		a	-1.05 / 20.71		a	-3.73 / 21.08		a	-3.65 / 28.17		a
	w	0	1.35 / 0.49	a	a	1.30 / 0.54	a	a	1.27 / 0.47	a	a	1.26 / 0.31	a	a
		3	1.30 / 0.47	a	a	1.25 / 0.50	a	a	1.28 / 0.38	a	a	1.16 / 0.34	a	a
		6	1.26 / 0.31	a	a	1.20 / 0.53	a	a	1.29 / 0.53	a	a	1.23 / 0.24	a	a
		9	1.30 / 0.37	a	a	1.26 / 0.50	a	a	1.19 / 0.47	a	a	1.24 / 0.25	a	a
		12	1.27 / 0.24	a	a	1.25 / 0.42	a	a	1.33 / 0.49	a	a	1.16 / 0.25	a	a
		%	-0.18 / 13.50		a	-2.58 / 25.04		a	-3.51 / 20.62		a	-6.14 / 31.01		a
	m	0	1.07 ± 0.26	a	a	0.95 ± 0.13	a	a	1.08 ± 0.24	a	a	1.00 ± 0.14	a	a
		3	1.00 ± 0.24	a	a	0.94 ± 0.21	a	a	1.03 ± 0.18	a	a	0.98 ± 0.10	a	a
		6	1.03 ± 0.22	a	a	0.90 ± 0.22	a	a	1.05 ± 0.22	a	a	0.98 ± 0.18	a	a
		9	0.98 ± 0.16	a	a	0.91 ± 0.18	a	a	1.05 ± 0.18	a	a	0.98 ± 0.27	a	a
		12	1.04 ± 0.20	a	a	1.00 ± 0.16	a	a	1.05 ± 0.21	a	a	0.97 ± 0.13	a	a
		%	-0.85 ± 11.65		a	5.88 ± 13.06		a	-0.850 ± 11.65		a	-2.77 ± 14.69		a
**Se/Cu**	all	0	0.08 / 0.02	a	a	0.08 / 0.03	a	a	0.07 / 0.02	a	a	0.07 / 0.02	a	a
		3	0.08 / 0.02	a,b	a	0.08 / 0.03	a	a	0.07 / 0.03	a,b,c	a	0.08 / 0.03	a	a
		6	0.09 / 0.03	b	a	0.09 / 0.02	a	a	0.08 / 0.03	b	a	0.07 / 0.03	a	a
		9	0.09 / 0.02	b	a	0.08 / 0.03	a	a	0.08 / 0.02	b,c	a	0.08 / 0.04	a	a

12	0.08 / 0.02	a,b	a	0.09 / 0.02	a	a	0.07 / 0.02	a,c	a	0.07 / 0.04	a	a
%	-4.76 / 15.23		a	-2.15 / 16.72		a	-4.10 / 27.49		a	-3.26 / 32.81		a

WD = omnivores, Flex = flexitarians, VG = vegetarians, VN = vegans, w = women, m = men, t = time points of the study in month, % = percentual change after 12 months compared to baseline, GPX = glutathione peroxidase, CP = ceruloplasmin, Cu/Zn= copper/zinc ratio, Se/Cu = selenium/copper ratio

Results are presented as mean ± SD or Median / IQR

* Diet groups that do not share indices differ significantly (ordinary one-way ANOVA or Kruskall-Wallis test followed by Benjamini-Hochberg correction ($p < 0.05$))

† Within diet groups: time points that do not share indices differ significantly (repeated measures ANOVA or Friedman test followed by Benjamini-Hochberg correction ($p < 0.05$)).

$p < 0.05$)

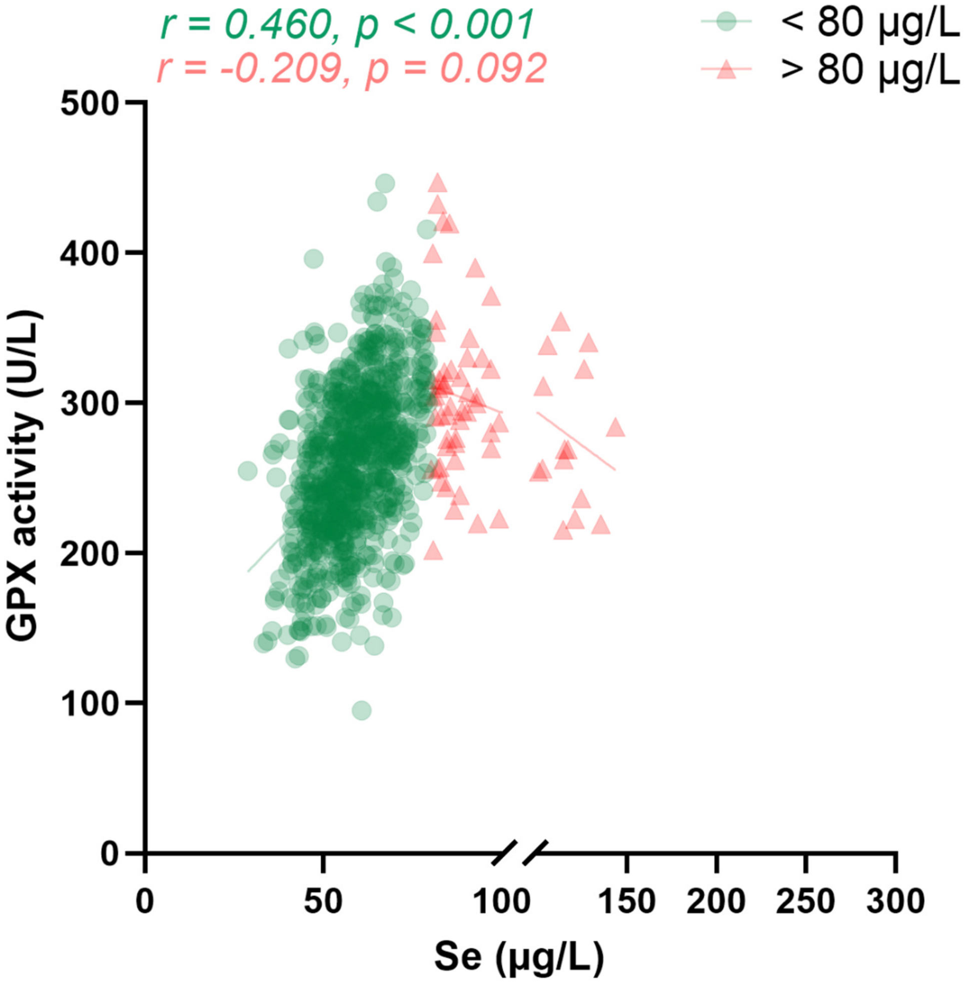

Figure S1. Correlation analysis of total serum selenium (Se) and glutathione peroxidase (GPX) activity over the entire study period. GPX activities were divided into two groups based on serum Se concentration < 80 μg/L (green) and > 80 μg/L (red). Correlation analysis was performed based on Spearman correlation ($p < 0.05$).

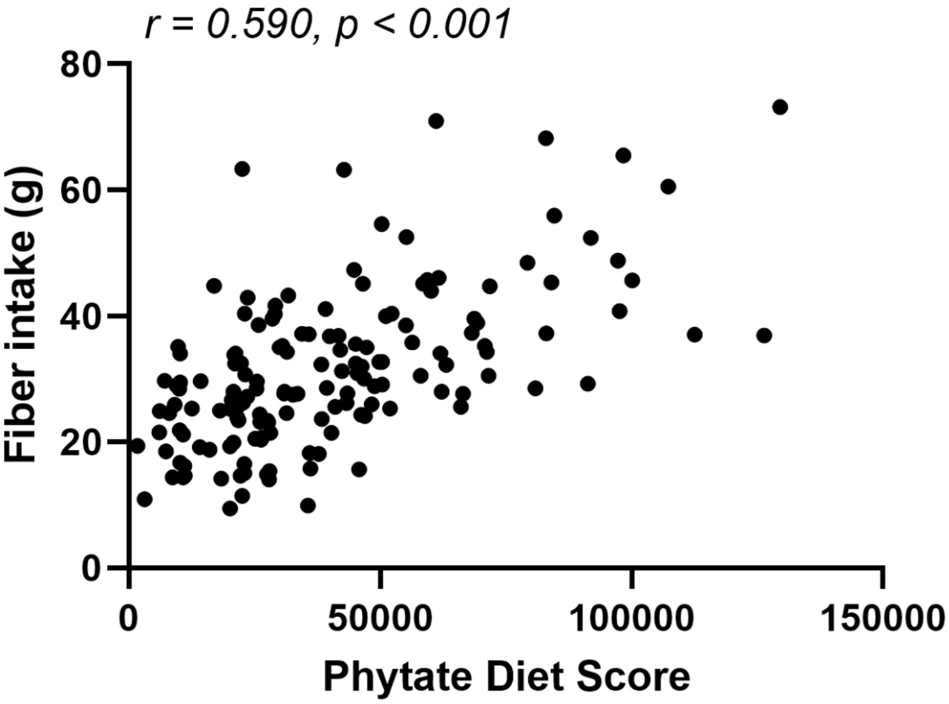

Figure S2. Correlation analysis of total daily fiber intake and Phytate Diet Score. Correlation analysis was performed based on Spearman correlation ($p < 0.05$).
